# Influence of Glutathione S-transferase Mu 1 (GSTM1), Glutathione S-transferase Theta 1 (GSTT1), and N-acetyltransferase 2 (NAT2) Variants on Bladder Cancer Progression and Recurrence Rating: A Bosnian and Herzegovinian Case-Control Study

**DOI:** 10.7759/cureus.91948

**Published:** 2025-09-09

**Authors:** Osman Hadziosmanovic, Benjamin Kulovac, Amina Valjevac, Almir Fajkić, Izabela Uzar, Grazyna Adler

**Affiliations:** 1 Department of Urology and Kidney Transplant, Clinical Center University of Sarajevo, Sarajevo, BIH; 2 Department of Human Physiology, University of Sarajevo, Faculty of Medicine, Sarajevo, BIH; 3 Department of Pathophysiology, University of Sarajevo, Faculty of Medicine, Sarajevo, BIH; 4 Department of General Pharmacology and Pharmacoeconomics, Pomeranian Medical University, Szczecin, POL; 5 Department of Studies in Antropogenetics and Biogerontology, Pomeranian Medical University, Szczecin, POL

**Keywords:** bladder cancer, genetic polymorphisms, gst, prognostic markers, snps

## Abstract

Introduction: It is suggested that bladder cancer (BC) development is linked to glutathione S-transferase (GST) enzymes. This study aimed to determine the correlation between glutathione S-transferase Mu 1 (GSTM1), glutathione S-transferase Theta 1 (GSTT1), and N-acetyltransferase 2 (NAT2) variants with BC progression and recurrence rating.

Materials and methods: This study included 105 Bosnian and Herzegovinian subjects: 60 patients with histopathologically confirmed BC and 45 controls without urological diseases. GSTM1, GSTT1 (rs36631 and rs17856199, respectively), and NAT2 (rs1799929, rs1799930, and rs1799931) were investigated.

Results: Both one- and five-year probabilities of progression were not significantly different in GSTM1 and NAT2 polymorphisms. One-year probability of progression was significantly higher in the GSTT1 T-- (null) than the T++ (wildtype) genotype (14.7% (±6.9) vs. 8.9% (±6.7), respectively; p=0.048). Five-year probability of progression was significantly higher in the GSTT1 T-- than the T++ genotype (39.4% (±14.7) vs. 25.5% (±16.6), respectively; p=0.045). THE GSTT1 T-- genotype was an independent predictor in the one-year probability of recurrence and progression (p=0.03 and p=0.01, respectively). GSTT1 T-- genotype and age were independent predictors for the five-year probability of recurrence (p=0.032 and p=0.04, respectively) as well as independent predictors of the five-year probability of progression (p=0.012 and p=0.03, respectively).

Conclusions: The GSTT1 T-- genotype was an independent predictor in the one- and five-year probabilities of both recurrence and progression of BC. GSTT1 rs17856199 may be a significant factor in the development of tumors and the course of disease in Bosnian and Herzegovinian BC patients.

## Introduction

Bladder cancer (BC) is a major global health burden, with ~400,000 new cases and ~150,000 deaths annually. The source of BC is the bladder's transitional epithelial lining (known as urothelium). Transitional cell cancer (TCC) accounts for approximately 90% of BC cases, while small cell carcinoma, adenocarcinoma, and squamous cell carcinoma are less common bladder malignancies that have originated from distinct cells (neuroendocrine, glandular epithelial, and squamous epithelial cells, respectively) [1].

It is suggested that enzymes known as glutathione S-transferases (GSTs) are significant for the detoxification of xenobiotics, including cancer-causing substances: monohalomethanes employed as solvents, methylating agents, and insecticides [2]. The capacity to obtain substrates has an impact on the body's detoxification of carcinogens, exposing cells to harmful compounds, damaging DNA, and raising the risk of tumor development [3]. The GSTs are enzymes that belong to a group encoded by many genes; among them, the cytosolic GSTs account for 10% of total cellular proteins [4]. Among genes encoding the cytosolic classes are glutathione S-transferase Mu 1 (GSTM1) and glutathione S-transferase Theta 1 (GSTT1), related to Mu and Theta enzymes, respectively. The genes mentioned above are polymorphic, with numerous variants, including rs36631 and rs17856199, which are linked to three alleles of GSTM1, two functional and one null allele (M-), representing a complete gene deletion that results in the absence of GSTM1 enzyme activity. Similarly, the GSTT1 gene has one functional allele and one null allele (T-), with the null variant abolishing enzyme function. It is suggested that the complete null genotype (M--T--) is linked to a lack of enzyme and BC [5,6]. Ha et al. have demonstrated a correlation between aggressive BC features and GSTT1 [7]. Moreover, according to Hireche et al., smoking and the GSTM1 M--, GSTT1 T-- (double null genotypes), separately were risk factors for BC in the Algerian population [8].

The N-acetyltransferase 2 gene (NAT2) encodes the NAT2 isozyme, which deactivates carcinogens, including hydrazines and aromatic, heterocyclic amines. There have been reports linking the NAT2 variants to drug-induced toxicities and a range of cancers [9-12]. Although allele frequencies of the NAT2 gene differ widely across ethnicities, allele *4 is regarded as the wildtype, rapid allele [13], while allele *5 (rs1799929), allele *6 (rs1799930), and allele *7 (rs1799931) reduce enzyme activity and have been considered slow alleles [13]. Subjects carrying two rapid alleles are rapid acetylators, those carrying one rapid allele and one slow allele are intermediate acetylators, and those carrying two slow alleles are slow acetylators. The genotypes can also be divided into two groups depending on whether at least one *4 allele is present or not.

Recently, a few studies have examined the correlation between the NAT2 variants and the risk of BC, but results on the impact of the slow acetylator genotype on BC were inconsistent [14,15]. Furthermore, it was suggested to study these in different ethnic groups; the very slow acetylator NAT2 *6*6 genotype was associated not only with the risk of developing BC but also with an increased risk for progression and recurrence [14]. A recent meta-analysis on the association of NAT2 variants with BC risk in a Chinese population included 10 studies with 896 BC subjects and 1188 controls and showed that slow acetylators had a significantly higher risk for BC [15].

Tobacco smoking remains the major risk factor, with evidence suggesting higher susceptibility among slow acetylators, though results are inconsistent [16-19]. However, the association was significant when smoking intensity and BC risk were assessed. Although there is broad knowledge on variants of GSTM1, GSTT1, and NAT2, there are contradictory reports on their relationship with BC in different populations [20]. Despite extensive research, data on GSTM1, GSTT1, and NAT2 variants in BC remain inconsistent across populations, and no studies have yet addressed this association in the Bosnian and Herzegovinian population.

Hence, this study aimed to determine the association between GSTM1, GSTT1, and NAT2 variants and BC progression and recurrence rating in the Bosnian and Herzegovinian population. We hypothesized that the GSTT1 T-- and GSTM1 M-- genotypes, as well as slow acetylator NAT2 variants (*5, *6, *7), would be associated with an increased likelihood of recurrence and progression in patients diagnosed with BC.

## Materials and methods

Subjects

This was a case-control study with prognostic modelling, in which genotype distributions were compared between BC cases and controls. Importantly, the one- and five-year probabilities of recurrence and progression were not directly observed but estimated using the European Organisation for Research and Treatment of Cancer (EORTC) risk tables based on baseline tumor characteristics and were analyzed as prognostic model outputs. Among 105 subjects, 60 were patients with histopathologically confirmed BC and 45 controls without manifest signs of urological diseases, such as hematuria, dysuria, flank pain, urinary frequency, or recurrent urinary tract infections. In both groups, the examination process included demographics, history of active and passive smoking, and dietary habits.

The study and all analyses were conducted at the Department of Urology, Clinical Center University of Sarajevo, Sarajevo, Bosnia and Herzegovina. All procedures performed in the studies were conducted according to the standards of the Declaration of Helsinki (1975, revised 2000). The study was approved by the Local Ethics Committee of the Clinical Center University of Sarajevo (approval number: 0604-94630; date: 01.02.2023). Informed consent was obtained from all individual participants included in the study.

Inclusion criteria in the study group were age above 18 years and pathohistologically confirmed BC. The exclusion criteria for both groups were as follows: a benign neoplasm of the bladder, previously confirmed cancer of other organs, and missing socio-epidemiological data. All study participants were residents of Sarajevo and the surrounding areas, which are not considered endemic regions for Balkan endemic nephropathy (BEN).

Medical examination and diagnostic workup

The initial selection included subjects with suspected BC and included hematuria, unexplained symptoms from the lower part of the urinary tract, and urine cytology suspicious for malignancy. The physical examination and the usual laboratory workup, an ultrasound examination of the urinary bladder and abdomen, as well as a CT urography, were performed. The BC was confirmed by cystoscopy; all macroscopic features of the tumor (localization, size, and appearance) and mucosal irregularities were described. Complete diagnostic processing was carried out in the institutions of the Clinical Center University of Sarajevo. Additional prognostic variables, including one-year and five-year probabilities of recurrence and progression, were calculated using the EORTC risk tables based on baseline tumor characteristics (number of tumors, tumor size, prior recurrence rate, T category, presence of carcinoma in situ, and tumor grade). These values represent validated prognostic estimates and were not derived from actual longitudinal follow-up.

Treatment

All patients received standard treatment in compliance with international guidelines. Non-muscle-invasive cases were treated with transurethral resection of the bladder (TUR), followed by intravesical instillations that could include Bacillus Calmette-Guérin (BCG), clinically indicated. Muscle-invasive cases were managed by TUR of the bladder followed by radical cystectomy or systemic therapy, e.g., platinum-based chemotherapy, depending on staging details and comorbidity profile as well as suitability of the patient for the treatment. The institutional multidisciplinary tumor board made all treatment decisions.

Pathohistological processing of the preparation

Bladder tumor tissue obtained by TUR was processed using the standard histological technique with paraffin blocks and stained with hemalaun-eosin. The pathological description included the following: the histological type and subtype of the tumor, the degree of malignancy (differentiation) of the tumor ("low grade" or "high grade") or grading from GX to G4, the status of the subepithelial connective tissue, the muscle layer of the mucous membrane, lymphocapillary invasion, and involvement of the muscle layer bladder walls.

DNA extraction and genotyping

Each study participant underwent a venous blood sampling into the vials with ethylenediaminetetraacetic acid (EDTA). The DNA extraction was performed using the QIAamp® DNA kit, according to the manufacturer's instructions (Fifth Edition, QIAGEN, Hilden, Germany). The DNA samples were stored at 4°C until analyzed.

The single-nucleotide polymorphism (SNP) GSTM1 rs36631 was determined by the multiplex polymerase chain reaction (PCR) method using High-Capacity cycler Cycler-Technology for Life (SensoQuest, Gottingen, Germany), and GSTM1 variants were tested using the relevant primers, as follows: forward: 5'GAA CTC CCT GAA AAG CTA AAG C3' and reverse: 5'GTT GGG CTC AAA TAT ACG GTG G3' (PCR product: 219 bp, TIB Mol Biol, Poznan, Poland). For PCR, the internal reference gene, HBB (known as hemoglobin β, β-globin), was used. The HBB was determined using the primers as follows: forward: 5'GAA GAG CCA AGG ACA GGT AC3' and reverse: 5'CAA CTT CAT CCA CGT TCA CC3' (PCR product: 268 bp). The PCR analysis was performed under the following conditions: 10 minute denaturation at 95°C, then 40 cycles during one minute at 95°C, one minute at 56°C, one minute at 72°C, and final extension for 10 minutes at 72°C. The PCR products were determined by electrophoresis in a 3% agarose gel (Agarose DNA Grade Electran) and visualized with DNA-star dye (Lonza, Inc., Rockland, Maine, United States).

Three NAT2 SNPs, namely, rs1799929 (481C>T, allele *5), rs1799930 (590G>A, allele *6), and rs1799931 (857G>A, allele *7), were detected using real-time PCR on the Roche LightCycler® 2.0 (Roche, Bazylea, Switzerland).

PCR of selected variants was performed using the StepOne™ Real-Time PCR System (Roche Diagnostics, Warsaw, Poland), TaqMan Genotyping Master Mix, and commercial pre-designed TaqMan SNP Genotyping Assays (Assay ID: C_1204092_20, C_12040091_10, and C_572770_20 for alleles *5, *6, and *7, respectively) (Thermo Fisher Scientific, Waltham, Massachusetts, United States), according to the manufacturer's instructions.

Before amplification, the samples were heated for 10 minutes at 95°C, followed by amplification: 40 cycles in two PCR steps at 95°C for 15 seconds and 60°C for one minute. Data were analyzed with TaqMan Genotyper Software v. 1.0.1. Approximately 10% of the samples were re-genotyped to monitor genotyping quality, and the same results were obtained.

All genetic investigations were carried out at the Department of Studies in Antropogenetics and Biogerontology, Pomeranian Medical University, Szczecin, Poland.

Statistical analysis

Statistical analysis of the data was conducted using the SPSS (Statistical Package for Social Sciences) computer program, version 13.0 (SPSS Inc., Chicago, Illinois, United States). The Kolmogorov-Smirnov and Shapiro-Wilk tests were used to determine whether the continuous variables followed a normal distribution. The results of the research are presented as mean value (X) and standard deviation (SD) for variables that follow a normal distribution. The comparison of mean values between groups was performed with the Student t-test for independent samples for variables with a normal distribution. Evaluation of categorical variables was performed with the chi-squared test or Fisher's test. Allele frequency was calculated by counting genotypes. The observed number of each genotype was compared with the expected distribution of genotypes following the Hardy-Weinberg equilibrium using the chi-squared test. The chi-squared test was also used to examine the differences in the frequency of polymorphisms between patients and healthy subjects. The risk of developing a disease was assessed using logistic regression, and the outcomes were adjusted for sex and age. Given the small sample size, results should be interpreted with caution as hypothesis-generating. Probabilities of recurrence and progression at one and five years, derived from the EORTC risk tables as described above, were analyzed as continuous outcomes. As these values are modelled estimates based on baseline tumor characteristics rather than observed longitudinal data, analyses were interpreted in the context of their prognostic rather than descriptive nature. Given the exploratory nature of the study and the limited sample size, no formal correction for multiple comparisons was applied; therefore, p-values are presented as descriptive indicators of statistical association rather than confirmatory evidence. All tests were two-tailed, and a p-value of <0.05 was considered statistically significant.

## Results

Baseline demographic and clinical characteristics of the BC patients and control subjects are summarized in Table 1.

**Table 1:** Baseline characteristics of patients with BC and control subjects Values are presented as mean±standard deviation and percentage. *EORTC recurrence and progression probabilities are derived from the EORTC risk tables based on baseline tumor characteristics; these are modelled estimates, not observed follow-up outcomes. N: number of subjects; BC: bladder cancer; CT: computed tomography; EORTC: European Organisation for Research and Treatment of Cancer; SD: standard deviation; NA: not applicable due to measures relevant only for BC patients

	BC patients (N=60)	Controls (N=45)
Age, years (±SD)	70.1 (±9.9)	65.5 (±9.96)
Males/females, n (%)	52 (86.7)/8 (13.3)	35 (77.8)/10 (22.2)
Smoking	46 (75.4)	36 (610)
Positive CT findings, n (%)	50 (83.3)	NA
Cancer grade, n (%)		
Low grade	16 (26.7)	-
High grade	44 (73.3)	-
Number of cancer lesions	1.6 (±1.0)	-
Size of the cancer	2.8 (±2.4)	-
Recurrence, n (%)	41 (68.3)	NA
Cancer stage, n (%)		
pTa	15 (25)	-
pT1	28 (46.7)	-
pT2	14 (23.3)	-
pT3	2 (3.3)	-
pT4	1 (1.7)	-
Cancer type, n (%)		
Non-muscle-invasive cancer	43 (71.7)	-
Muscle-invasive cancer	17 (28.3)	-
EORTC risk scores*		
Recurrence risk score	5.5 (±3.0)	-
Recurrence risk, n (%)		
Low	4 (9.3)	-
Intermediate	43 (79.1)	-
High	5 (11.6)	-
1-year probability of recurrence	35 (±12.5)	-
5-year probability of recurrence	56.9 (±12.7)	-
Progression risk score	9.1 (±4.5)	-
Progression risk, n (%)		
Low	4 (9.3)	-
Intermediate	28 (65.1)	-
High	11 (25.6)	-
1-year probability of progression	9.9 (±6.9)	-
5-year probability of progression	27.8 (±17.0)	-

No statistically significant difference was observed in the frequency of the GSTM1 M-- genotype (rs36631) between BC patients and controls (OR=2.05; 95% CI: 0.94-4.5; p=0.073). Similarly, this association remained non-significant after adjusting for sex and age (adjusted OR=2.15; 95% CI: 0.95-4.9; p = 0.065). For the GSTT1 T-- genotype (rs17856199), no significant difference was observed between groups (OR=1.04; 95% CI: 0.38-2.8; p=0.94) nor in the adjusted model (adjusted OR=0.77; 95% CI: 0.26-2.2; p=0.63). Combined GSTM1/GSTT1 genotype analysis showed no significant association with BC presence. Compared to double wildtype (M++/T++), ORs were 1.1 (95% CI: 0.28-4.4; p=0.9) for M++/T--, 2.1 (95% CI: 0.87-4.9; p=0.1) for M--/T++, and 2.2 (95% CI: 0.5-10.0; p=0.3) for M--/T--. Regarding NAT2 polymorphisms, none of the tested genotypes were significantly associated with BC risk. For rs1799929 CT, OR was 1.2 (95% CI: 0.4-3.4; p=0.7); for rs1799930 GA and AA, ORs were 1.0 (95% CI: 0.4-2.3; p=1.0) and 1.6 (95% CI: 0.4-6.9; p=0.6); and for rs1799931 GA, OR was 1.5 (95% CI: 0.1-17.3; p=0.7). Adjusted ORs were similarly non-significant (Table 2).

**Table 2:** Genotype frequencies of GSTM1, GSTT1, and NAT2 and their association with BC risk Values are presented as number (percentage); p-value in two-sided chi-squared test. *Adjusted for sex and age GSTM1: glutathione S-transferase Mu 1; GSTT1: glutathione S-transferase Theta 1; NAT2: N-acetyltransferase 2; CI: confidence interval; OR: odds ratio; WT: wildtype; NT: nulltype; NA: not available; BC: bladder cancer

	Case n (%)	Control n (%)	Unadjusted OR (95% CI)	P- value	Adjusted* OR (95% CI)	P-value
GSTM1						
rs36631 M1++ (WT)	24 (40)	26 (57.8)	1.0 (ref)	-	1.0 (ref)	-
M1-- (NT)	36 (60)	19 (42.2)	2.05 (0.94-4.5)	0.073	2.15 (0.95-4.9)	0.065
GSTT1 rs17856199						
T1++ (WT)	49 (81.7)	37 (82.2)	1.0 (ref)	-	1.0 (ref)	-
T1-- (NT)	11 (18.3)	8 (17.8)	1.04 (0.38-2.8)	0.94	0.77 (0.26-2.2)	0.63
GSTM1/GSTT1						
M++/T++	19 (31.7)	21 (46.7)	1.0 (ref)	-	1.0 (ref)	-
M++/T--	5 (8.3)	5 (11.1)	1.1 (0.28-4.4)	0.9	0.8 (0.2-3.4)	0.7
M--/T++	30 (50)	16 (35.6)	2.1 (0.87-4.9)	0.1	2.1 (0.9-5.2)	0.1
M--/T--	6 (10)	3 (6.7)	2.2 (0.5-10.0)	0.3	1.7 (0.4-8.2)	0.5
NAT2						
rs1799929 (481C>T, allele *5)						
CC	49 (81.7)	38 (84.4)	1.0 (ref)	-	1.0 (ref)	-
CT TT	11 (18.3) 0	7 (15.6%) 0	1.2 (0.4-3.4) NA	0.7 NA	1.0 (0.3-3.0) NA	0.99 NA
rs1799930 (590G>A, allele *6)						
GG	32 (53.3)	25 (55.6)	1.0 (ref)	-	1.0 (ref)	-
GA	22 (36.7)	17 (37.8)	1.0 (0.4-2.3)	1.0	1.2 (0.5-2.8)	0.7
AA	6 (10)	3 (6.7)	1.6 (0.4-6.9)	0.6	1.7 (0.4-7.9)	0.5
rs1799931 (857G>A, allele *7)						
GG	58 (96.7)	44 (97.8)	1.0 (ref)	-	1.0 (ref)	-
GA AA	2 (3.3) 0	1 (2.2) 0	1.5 (0.1-17.3) NA	0.7 NA	1.9 (0.2-22.0) NA	0.6 NA

Carriers of the GSTT1 T-- genotype had a significantly higher estimated probability of progression at both one year (14.7% ±6.9 vs. 8.9% ±6.7; p=0.048) and five years (39.4% ±14.7 vs. 25.5% ±16.6; p=0.045) compared to T++ carriers. No statistically significant differences in progression probabilities were found for different GSTM1 or NAT2 genotypes (Table 3).

**Table 3:** Association between GSTM1, GSTT1, and NAT2 genotype frequencies and progression risk in BC patients Values are presented as mean±standard deviation. *Probabilities represent modelled estimates derived from the EORTC risk tables, based on baseline tumor characteristics and are not observed outcomes. GSTM1: glutathione S-transferase Mu 1; GSTT1: glutathione S-transferase Theta 1; NAT2: N-acetyltransferase 2; WT: wildtype; NT: nulltype; BC: bladder cancer; EORTC: European Organisation for Research and Treatment of Cancer

	Progression risk score	P-value	1-year probability of progression* (%)	P-value	5-year probability of progression* (%)	P-value
GSTM1						
WT M++	9.5 (±3.9)	0.6	10.1 (±6.8)	0.84	28.6 (±16.5)	0.8
NT M--	8.8 (±4.9)		9.7 (±7.1)		27.2 (±17.6)	
GSTT1						
WT T++	8.6 (±4.4)	0.11	8.9 (±6.7)	0.048	25.5 (±16.6)	0.045
NT T--	11.6 (±4.4)		14.7 (±6.9)		39.4 (±14.7)	
NAT2						
rs1799929 (481C>T, allele *5)						
CC	8.9 (±4.5)	0.47	9.8 (±6.9)	0.86	27.5 (±17.1)	0.83
CT and TT	10.3 (±4.8)		10.3 (±7.4)		29.2±17.8	
rs1799930 (590G>A, allele *6)						
GG	9.5 (±4.4)	0.57	10.0 (±6.9)	0.85	28.3 (±17.1)	0.85
GA and AA	8.7 (±4.6)		9.7 (±7.0)		27.3 (±17.3)	
rs1799931 (857G>A, allele *7)						
GG	5.4 (±1.4)	0.44	14.6 (±6.5)	0.76	36.7 (±12.8)	0.72
GA	4.0 (±1.4)		12.1 (±5.8)		34.0 (±11.1)	

In the multivariate regression model, the GSTT1 T-- genotype was identified as an independent predictor for one-year recurrence (B=0.10; 95% CI: 0.10-0.62; p=0.03) and progression (B=0.15; 95% CI: 0.19-0.63; p=0.01) (Table 4). Additionally, both GSTT1 T-- and older age were independent predictors for five-year recurrence (GSTT1: B=0.12; 95% CI: 0.10-0.58; p=0.032; age: B=-0.004; 95% CI: -0.008 to -0.07; p=0.04) and five-year progression (GSTT1: B=0.20; 95% CI: 0.14-0.52; p=0.012; age: B=-0.006; 95% CI: -0.01 to -0.001; p=0.03) (Table 5). No significant associations were found between smoking intensity and BC risk across any of the tested genotypes (all regression coefficients close to zero with wide 95% CI overlapping unity; data not shown).

**Table 4:** Linear regression analysis between GSTT1 T-- and the probability of recurrence and progression after one year in BC patients Values are regression coefficients (B)±standard error (SE), standardized beta (ß), p-value, and 95% confidence interval (CI) for B. Probabilities represent modelled estimates from the EORTC risk tables, not observed outcomes. GSTT1: glutathione S-transferase Theta 1; WT: wildtype; NT: nulltype; BC: bladder cancer; EORTC: European Organisation for Research and Treatment of Cancer

	1-year probability of recurrence					1-year probability of progression				
	B	SE	ß	p	(95% CI) for B	B	SE	ß	p	(95% CI) for B
Constant	0.6	0.2	-	0.001	0.25 to 0.90	0.26	0.09	-	-	0.09 to 0.40
Age	-0.003	0.002	-0.28	0.8	-0.007 to 0.000	-0.002	0.001	-0.32	0.035	-0.004 to 0.000
Male	-0.002	0.05	-0.005	0.98	-0.11 to 0.10	-0.01	0.29	-0.057	0.7	-0.07 to 0.05
GSTT1 Nulltype T--	0.1	0.05	0.34	0.03	0.10 to 0.62	0.15	0.28	0.4	0.01	0.19 to 0.63

**Table 5:** Linear regression analysis between GSTT1 T-- and the probability of recurrence and progression after five years in BC patients Values are regression coefficients (B)±standard error (SE), standardized beta (ß), p-value, and 95% confidence interval (CI) for B. Probabilities represent modelled estimates from the EORTC risk tables, not observed outcomes. GSTT1: glutathione S-transferase Theta 1; WT: wildtype; NT: nulltype; BC: bladder cancer; EORTC: European Organisation for Research and Treatment of Cancer

	5-year probability of recurrence					5-year probability of progression				
	B	SE	ß	p	(95% CI) for B	B	SE	ß	p	(95% CI) for B
Constant	0.8	0.16	-	0.001	0.5 to 1.2	0.7	0.2	-	0.002	0.3 to 1.1
Age	-0.004	0.002	-0.32	0.04	-0.008 to 0.07	-0.006	0.003	-0.3	0.03	-0.01 to 0.001
Male	-0.009	0.054	-0.03	0.87	-0.12 to 0.10	-0.02	0.07	-0.05	0.75	-0.2 to 0.12
GSTT1 Nulltype T--	0.12	0.052	0.34	0.032	0.1 to 0.58	0.2	0.07	0.39	0.012	0.14 to 0.52

## Discussion

Our findings suggest that while GSTM1 and NAT2 polymorphisms were not associated with BC susceptibility in our cohort, the GSTT1 T-- genotype showed a significant and independent association with one- and five-year risk of disease progression and recurrence.

It is reported that variations in the enzymes' activity responsible for detoxifying substances could work together to affect the amount of DNA damage and the risk of the occurrence of diseases, including BC. Carrying the GSTM1 M-- and GSTT1 T-- genotypes has been associated with a higher risk of developing BC due to their reduced ability to metabolize specific toxins. In contrast, the NAT2 slow genotype increased the risk of BC in female ever smokers [21,22].

Our results have shown no differences between the frequency of both GSTM1 M-- and none for NAT2 genotypes in BC patients compared to healthy subjects. However, the one- and five-year probabilities of progression were significantly higher in the GSTT1 T-- than the wildtype genotype. Although the GSTT1 T-- genotype was not associated with an increased risk of bladder cancer occurrence, it emerged as a significant prognostic marker. Similar findings have been reported in previous studies, suggesting that some genetic variants may influence disease behavior rather than susceptibility [7].

In a meta-analysis by Yu et al., 12,369 patients and 15,333 healthy subjects were analyzed. In the entire group of patients, the findings showed a significant correlation between the GSTT1 T-- genotype and BC susceptibility. In terms of correlation across ethnic groups, only Caucasians showed significant relationships between the GSTT1 T-- genotype and BC [23].

The smoking and the GSTT1 T-- genotype were found to be substantially correlated with BC in the Korean case-control study, while the GSTM1 M-- genotype was not [24]. On the one hand, numerous studies have linked GSTT1 T-- to an increased risk of BC in many populations; on the other, they have linked TCC to the fully functioning, GSTT1 T++ genotype [5,25,26].

After stratification of patients according to smoking status, our results did not show a significant association between smoking and BC in any of the tested genotypes. There was also no significant association between smoking intensity and BC in any of the tested genotypes. The lack of association between smoking and bladder cancer in our cohort may reflect underreporting, exposure misclassification, or limited sample size. While smoking is a well-established risk factor for bladder cancer, these methodological constraints may have masked its effect in this study.

Given that the GSTT1 T-- genotype predicted both short- and long-term recurrence and progression independently of smoking status, this marker may hold potential as a prognostic tool in clinical risk stratification of BC patients in this region.

Contradictory results may be linked to both diverse exposure in various populations to xenobiotics and different prevalence of GSTM1, GSTT1, and NAT2 variants. In an extensive systematic review, the highest prevalence of GSTM1 M-- was observed in European and Middle Eastern healthy subjects (50% and 48%, respectively), followed by Native American subjects (36%), and the lowest prevalence was found in the sub-Saharan African healthy population (27%), while the highest prevalence of GSTT1 T-- was in sub-Saharan African healthy subjects, 32%, through 25% in the Middle Eastern healthy population, and the lowest and similar in European and Native American, 17% and 14%, respectively [27]. 

Considering the NAT2 variants by 1,000 genomes, the highest frequency of rs1799929 *5 was in the European population (44%) and the lowest in East Asia subjects (4%). The highest frequency of rs1799930 *6 was observed in Caucasians, whereas the lowest frequency was found in the American population, at 36% and 17%, respectively. Moreover, rs1799931 *7 was most frequently observed in the East Asian population (18%) and least common in the Caucasian population (1%) [27]. 

It is currently unclear which carcinogens are substrates of particular GST enzymes and whether the products of glutathione conjugation induce carcinogenesis in humans. Results of tests conducted on animals confirmed that the functions of particular GST enzymes in the biotransformation of bladder carcinogens require more research. Furthermore, it is reported that low-functioning genetic variants of GSTM1 and GSTP1 (glutathione S-transferase Pi 1) can interact with the GSTT1 variants and affect the risk of TCC in people [28]. The GSTM1-- and GSTT1 T-- genotype has been linked to an increased risk of BC, as demonstrated by Yu et al. [22]. In the regression analysis model, it was shown that the GSTT1 T-- genotype was an independent predictor of one-year probability of recurrence (OR: 0.34; 95% CI: 0.1-0.62) as well as an independent predictor of one-year probability of progression (OR=0.4; 95% CI: 0.19-0.63). The GSTT1 T-- genotype and age were independent predictors of the five-year probability of recurrence (OR=0.34; 95% CI: 0.1-0.58) as well as independent predictors of the five-year probability of progression (OR=0.39; 95% CI: 0.14-0.52).

Goerlitz et al. received opposite results in the Egyptian population; they showed that functional polymorphisms in the GSTM1 and GSTT1 genes were not statistically significantly linked to BC and significant interactions between genotypes and smoking or gender also were not found [29].

In the Mongolian population, a significant correlation between tobacco users with the GSTM1 M-- genotype, NAT2 low acetylator phenotype, and BC was found [30]. Still, a recent study in the German population showed that NAT2 was not significantly linked to a higher risk of BC compared to healthy subjects [15]. We can conclude that in the Bosnian and Herzegovinian population, the GSTT1 T-- genotype might be an independent predictor of the one- and five-year probability of recurrence rating and BC progression. However, we concluded that subsequent studies with a larger sample size are required to validate the results and test their clinical utility. To our knowledge, no previous study has examined GSTM1, GSTT1, or NAT2 variants in the Bosnian and Herzegovinian population, either in bladder cancer or in other types of cancer. This highlights a broader gap in genetic epidemiology data from this region, reinforcing the exploratory and hypothesis-generating nature of our work.

This study has several limitations. First, the sample size was limited, particularly for subgroup analyses, and the study was underpowered for several comparisons. This small sample size reduces statistical power and limits the generalizability of our findings. Second, no correction for multiple comparisons was applied, which increases the risk of false-positive results and warrants cautious interpretation. Third, no data were collected on exposure to potential environmental carcinogens. We did not collect data on occupational exposure to carcinogens, which may have influenced bladder cancer risk estimates in our cohort. Fourth, treatment heterogeneity and lack of staging details may have influenced progression and recurrence estimates. Finally, findings were not validated in an independent cohort, further limiting generalizability. As this was a pilot study with a modest sample size, the results should be considered hypothesis-generating rather than definitive; however, the internal consistency of the prognostic signal across multiple endpoints supports the need for validation in larger, multicenter cohorts.

## Conclusions

Our findings indicate that the GSTT1 T-- genotype may be associated with higher EORTC model-predicted probabilities of recurrence and progression in Bosnian and Herzegovinian bladder cancer patients. Given the small sample size and cross-sectional design, these results should be considered preliminary and hypothesis-generating. Validation in larger, prospective cohorts is warranted to clarify their prognostic relevance. If confirmed, GSTT1 genotyping could be integrated into clinical risk stratification algorithms to identify patients at higher risk of recurrence and progression, enabling more intensive surveillance, tailored follow-up schedules, and potentially personalized therapeutic approaches in the Bosnian and Herzegovinian setting.
